# Aging Fibroblasts Adversely Affect Extracellular Matrix Formation via the Senescent Humoral Factor Ependymin-Related Protein 1

**DOI:** 10.3390/cells11233749

**Published:** 2022-11-24

**Authors:** Kento Takaya, Toru Asou, Kazuo Kishi

**Affiliations:** Department of Plastic and Reconstructive Surgery, Keio University School of Medicine, Tokyo 160-8582, Japan

**Keywords:** ependymin-related protein 1, skin aging, fibroblast, senescence-associated secretory phenotype, senescence

## Abstract

Skin senescence is characterized by a decrease in extracellular matrix and the accumulation of senescent fibroblasts in the dermis, and their secretion of humoral factors. Ependymin-related protein 1 (EPDR1) is involved in abnormal fibroblast metabolism and collagen deposition, however, its relation to skin aging is unclear. We investigated whether and how EPDR1 is involved in age-related dermal deterioration. When young dermal fibroblasts and senescent cells were co-cultured in a semipermeable membrane separation system, the young fibroblasts showed decreased gene expression of collagen type I α1 chain (*COL1A1*) and elastin, and increased expression of matrix metalloproteinase (*MMP*)*1* and *MMP3*. Senescence marker expression and EPDR1 production were increased in the culture medium of senescent cells. Treatment of young fibroblasts with recombinant EPDR1, enhanced matrix-related gene expression and suppressed *COL1A1* expression, whereas *EPDR1* knockdown had the opposite effects. EPDR1 gene and protein expression were increased in aged skin, compared to young skin. These results suggest that senescent cells affect nearby fibroblasts, in part through EPDR1 secretion, and exert negative effects on matrix production in the dermis. These results may lead to the discovery of potential candidate targets in the development of skin anti-aging therapies.

## 1. Introduction

Cellular senescence is a state of irreversible growth arrest caused by stress signals such as telomere shortening, oxidative damage, oncogene activation, and hypoxia [1]. Senescent cells have an enlarged cytoplasm, multiple nuclei, and different patterns of metabolism and gene expression, compared to proliferating cells [2]. They secrete various cytokines (senescence-associated secretory phenotype [SASP]) that affect the tissue microenvironment and disrupt tissue structure and function through paracrine effects [3,4]. This results in impaired tissue regeneration and contributes to age-related tissue damage, including impaired tissue homeostasis and tumorigenesis. López-Otín et al. [5] hypothesized that skin aging is attributed to the SASP caused by senescent cells accumulated in the epidermis and dermis, and the subcutaneous adipose tissue depot. The dermis is rich in extracellular matrix composed of collagen and elastin, which contribute to skin elasticity and surface morphology maintenance. Abnormal metabolism of the extracellular matrix by senescent cells causes skin aging. The amount of matrix decreases with age, leading to loss of skin elasticity, delayed wound healing, and changes in surface morphology [6,7].

The existence of senescence-inducing factors secreted by senescent cells has been demonstrated by heterochronic parabiosis experiments; when old and young mice were surgically connected and the blood was allowed to circulate between them, some tissues of the young mice developed aging-related phenotypes, such as delayed regeneration after injury [8,9]. Complement factor C1q [10] and growth differentiation factor 11 [11] have been identified as senescence-inducing factors; however, humoral factor-induced senescence in skin fibroblasts remains unclear.

We noted similar histological and biochemical changes in the wound repair of aged connective tissue and tissue affected by Dupuytren’s contracture [12]. In particular, there are metabolic abnormalities in the deposition of extracellular matrix proteins (especially collagen) and the presence of contractile myofibroblasts [13]. Genetic analysis of Dupuytren’s contracture revealed a marked elevation of ependymin-related protein 1 (EPDR1) gene expression [14], which reportedly contributes to excessive collagen contractility and fibroblast abnormalities [15].

Little is known about EPDR1. The protein is also known as mammalian ependymin-related protein 1 (MERP1) and as UCC1, which is upregulated in colorectal cancer [16]. Calcium-induced conformational changes in ependymin have been reported to be important for its interaction with the extracellular matrix, particularly with collagen [17,18]. Recent studies have shown that EPDR1 expression in beta cells is increased under metabolic overload (obesity) and directs glycolysis-derived pyruvate to the mitochondrial TCA cycle, thus enhancing stimulated secretory coupling and increasing insulin secretion [19]. Interestingly, through this mechanism, EPDR1 may reflect compensatory mitochondrial biosynthesis that replaces defective mitochondria, suggesting that its expression increased as a result of age-related cell organ dysfunction [20]. However, their function in the skin, particularly in the dermis, and their behavior during aging remain unresolved.

Activation of p16 INK4a expression has proven to be one of the most useful markers of senescence. As a cell cycle regulator, p16 INK4a limits cell cycle progression from G1 phase to S phase by inhibiting CDK4 and CDK6 (CDK4/6) kinases [21]. In addition, p16 INK4a expression is highly dynamic, barely detectable in healthy young tissues, but rapidly elevated in many tissues after aging or certain types of tissue damage [22,23,24]. Mouse studies suggest that accumulation of p16 INK4a leads to age-related loss of replication capacity in selected tissues, thereby causing some phenotypic aspects of aging [25].

Alternatively, the function of MMP in relation to the extracellular matrix is important for tracking aging; MMP have the ability to irreversibly degrade ECM components and shed the outer domains of cell surface receptors [26]. Numerous studies have linked cellular senescence to increased expression of MMP family members, which was also investigated in this study.

We aimed to investigate the role of EPDR1 in aging fibroblasts of the dermis, using a co-culture system in which young and aging dermal fibroblasts were separated by a semipermeable membrane to examine the association between EPDR1, and the effects of aging dermal fibroblasts on young dermal fibroblasts.

## 2. Materials and Methods

### 2.1. Cell Culture

Normal human dermal fibroblasts (NHDF; C-12300) were purchased from TakaraBio (Shiga, Japan). The cells were grown in low-glucose Dulbecco’s modified Eagle’s medium (DMEM; Wako Pure Chemical Industries, Osaka, Japan) supplemented with 10% fetal bovine serum (FBS; Thermo Fisher Scientific, Waltham, MA, USA) and 1% penicillin/streptomycin (Thermo Fisher Scientific). Fibroblasts exhibiting proliferative senescence were defined as having a population doubling level (PDL) of 50 or greater, and visually arrested proliferation as a result of consecutive passages over a 3-month period. Intracellular senescence-associated (SA)-β-gal activity was assessed using the Senescence β-Galactosidase Staining Kit from Cell Signaling Technology (Danvers, MA, USA), following the manufacturer’s introduction. To assess cell division, cells were incubated with bromodeoxyuridine (BrdU) at 37 °C for 24 and incubated with BrdU-FITC antibody (BrdUFlowEx FITC Kit; EXBIO Praha, a.s., Vestec, Czech Republic) for 30 min. Then, the cells were analyzed using a flow cytometer (BD Biosciences, Franklin Lakes, NJ, USA) and FlowJo (version 10.3).

### 2.2. Cell Co-Culture

Fibroblasts were cultured in DMEM containing 10% (*w*/*v*) FBS at 37 °C in humidified air with 5% CO_2_. UniWells Horizontal Co-Culture Plates (Ginreilab, Ishikawa, Japan) were used for co-culture. Senescent and young fibroblasts were seeded at a density of 1250 cells/cm^2^ into one of the two compartments. After two days, the fibroblasts and culture medium from both compartments were collected.

### 2.3. Immunocytochemistry

Cells were placed on glass slides and fixed in 4% paraformaldehyde at room temperature (20–25 °C) for 10 min. The cells were incubated with anti-EPDR1 antibody (PA5-140254, Thermo Fisher Scientific) and anti-p16ink4a antibody (ab211542; Abcam, Cambridge, UK) diluted 1:100 in phosphate-buffered saline (PBS) at 4 °C overnight. After three washes with PBS, the cells were incubated with Alexa Fluor 488-conjugated goat anti-rabbit antibody and Alexa Fluor 555-conjugated donkey anti-goat antibody (each from Thermo Fisher Scientific), diluted 1:2000 in PBS at room temperature for 1 h. After incubation, the slides were washed three times with PBS and counterstained for nuclear visualization using ProLong Gold Antifade Mountant (Thermo Fisher Scientific) containing 4′,6-diamidino-2-phenylindole.

### 2.4. Immunohistochemistry

Whole human skin fragments were collected from the trunks of healthy male volunteers (aged, 10- and 78-years). The volunteers had no obvious underlying diseases, no history of internal medicine use. The slides were deparaffinized by washing in xylene three times at room temperature (20–25 °C, 5 min per soak). Then, the slides were soaked twice in 100% ethanol (3 min per soak) and then stepwise in 95%, 80%, and 75% ethanol (3 min per soak), and rehydrated at room temperature. The slides were incubated with 3% goat serum in PBS at room temperature for 1 h to block nonspecific binding sites. The slides were then incubated with a 1:100 dilution of anti-EPDR1 antibody (PA5-140254, Thermo Fisher Scientific) in PBS at 4 °C overnight. After three washes with PBS, the slides were incubated with a 1:500 dilution of biotinylated rabbit anti-goat antibody (Vector Laboratories, Burlingame, CA, USA) in PBS at room temperature for 1 h. The signal was amplified by the avidin-biotinylated peroxidase complex (ABC) method using the VECTASTAIN ABC Kit (Vector Laboratories) and 20 mg/dL 3,3′-diaminobenzidine solution (FUJIFILM Wako Pure Chemicals, Osaka, Japan) for 1–3 min to develop color. Then, the sections were washed once with running tap water for 5 min before nuclear counterstaining with Gill’s hematoxylin solution (Merck Millipore, Billerica, MA, USA) at room temperature for 6 s. Finally, the sections were rinsed with tap water for 5 min, dehydrated in ethanol (twice in 95% and twice in 100%, 5 min per soak), rinsed with xylene three times, and sealed with Mount Quick Sealant (TaKaRa Bio, Shiga, Japan). The samples were observed using an integrated stereomicroscope (BZ-X800; Keyence, Osaka, Japan).

### 2.5. RNA Interference and Transfection Method

Cells were transfected with EPDR1 siRNA (122,437, 122,438, and 122,439; Silencer^TM^ siRNA, Thermo Fisher Scientific) using Lipofectamine 2000 (11668-019; Life Technologies, Invitrogen, Carlsbad, CA, USA). After 72 h, RNA was collected from the cells and specific gene knockdown was assessed using quantitative reverse transcription (RT-q) PCR.

### 2.6. Treatment of Dermal Fibroblasts with Recombinant Human Proteins

Young human skin fibroblasts (PDL 10-15) were maintained in DMEM containing 15 μg/mL recombinant EPDR1 (rEPDR1; ab162830, Abcam). The rEPDR1-containing medium was refreshed every 2 days. The cells were analyzed after 10 days. Cell viability after rEPDR1 intervention was measured using the MTT assay kit (CellQuanti-MTT^TM^ Cell Viability Assay Kit (CQMT-500, Bioassay Systems, Hayward, CA, USA)) according to the manufacturer’s protocol.

### 2.7. RNA Isolation, and Reverse Transcription

Total RNA was extracted from cells or skin tissues using a monophasic solution of phenol and guanidine isothiocyanate (ISOGEN; NipponGene, Tokyo, Japan) according to the manufacturer’s instructions. The RNA was mixed with a random primer, reverse transcriptase, and dNTP mixture (Takara Bio, Tokyo, Japan). The mixture was incubated in a T100TM thermal cycler (Bio-Rad Laboratories, Hercules, CA, USA) at 25 °C for 5 min, 55 °C for 10 min, and 80 °C for 10 min to heat-inactivate the reverse transcriptase and synthesize cDNA.

### 2.8. RT-qPCR

RT-qPCR was performed on an Applied Biosystems 7500 Fast Real-Time PCR System (Thermo Fisher Scientific). Briefly, 40 cycles of 95 °C for 3 s (denaturation) and 60 °C for 30 s (annealing and extension) were run, and the fluorescence of each sample was measured at the end of each cycle. In subsequent melting curve analysis, the temperature was increased from 60 °C to 95 °C and fluorescence was measured continuously. Gene expression was determined using primers for *EPDR1* (assay ID: Hs01556067_m1), *COL1A1* (Hs00164004_m1), elastin gene (*ELN)* (Hs00355783_m1), *MMP1* (Hs00899658_m1), *MMP3* (Hs00968305_m1), and *p16ink4a* (Hs00923894_m1) (all from Thermo Fisher Scientific) and a PCR master mix (Cat. No. 4352042; Applied Biosystems, Foster City, CA, USA) following the manufacturers’ instructions. *GAPDH* (Hs02786624_g1) was used as a reference gene for normalization. The gene expression level in the proliferating cell population was used as the baseline, and fold change values were determined by the 2^−ΔΔCT^ method.

### 2.9. Western Blotting

Total protein was extracted from cells using lysis buffer (50 mM Tris-HCl (pH 8.0), 150 mM NaCl, 0.5% Nonidet P-40, 0.5% sodium deoxycholate, and phenylmethylsulfonyl fluoride (all from FUJIFILM Wako Pure Chemical)). Equal volumes (40 μg) were electrophoresed on 10% polyacrylamide gels (Mini-PROTEAN TGX Precast Gels; Bio-Rad Laboratories) and transferred onto polyvinylidene difluoride membranes (Millipore, Bedford, MA, USA) using a Trans-Blot Turbo Transfer System (Bio-Rad Laboratories). After blocking with 3% nonfat milk at room temperature for 2 h, the membranes were incubated with primary antibodies against EPDR1 (PA5-140254; Thermo Fisher Scientific, 1:200), CDKN2A/p16INK4a (EPR1473; Thermo Fisher Scientific, 1:200), MMP9 (ab52631; Abcam, 1:2000), MMP3 (ab52915; Abcam, 1:1000), ELN (sc-58756; SantaCruz Biotechnology, Dallas, TX, USA, 1:500) and COL1A1 (GTX26308; GeneTex, Irvine, CA, USA, 1:200), diluted in blocking solution at 4 °C overnight. The next day, the samples were incubated with donkey anti-goat IgG H&L (HRP) (ab6885; Abcam) and goat anti-rabbit IgG H&L (HRP) (ab205718; Abcam) at a 1:1000 dilution at 37 °C for 2 h. After washing, immunoreactive protein bands were visualized using an Electrochemiluminescence Detection Kit (Pierce Biotechnology, Rockford, IL, USA). Images of the bands were acquired using a chemiluminescence imager (ImageQuant LAS 4000 mini; GE Healthcare, Chicago, IL, USA). Image analysis was performed using ImageJ (version 1.53p, National Institutes of Health, Bethesda, MA, USA). Each assay was repeated three times.

### 2.10. Enzyme-Linked Immunosorbent Assay (ELISA)

Senescent cells were prepared as described above, and the medium was changed to serum-free medium containing antibiotics. After 24 h, the conditioned medium was collected and EPDR1 expression was quantified using a Human Mammalian ependymin-related protein 1 (EPDR1) ELISA Kit (abx522130; Abbexa, Cambridge, UK), according to the manufacturer’s protocol.

### 2.11. Statistical Analysis

Statistical analyses were performed using GraphPad Prism (version 9; GraphPad Software, San Diego, CA, USA) or SPSS 22.0 (SPSS, Chicago, IL, USA). Mann–Whitney’s U test was used to analyze differences between the two groups. One-way analysis of variance and Tukey’s post hoc test were used to analyze differences among three or more groups. Statistical significance was set at *p* < 0.05.

## 3. Results

### 3.1. Senescent Fibroblasts Influence Extracellular Matrix Expression in Young Fibroblasts via Humoral Factors

**Figure 1:**
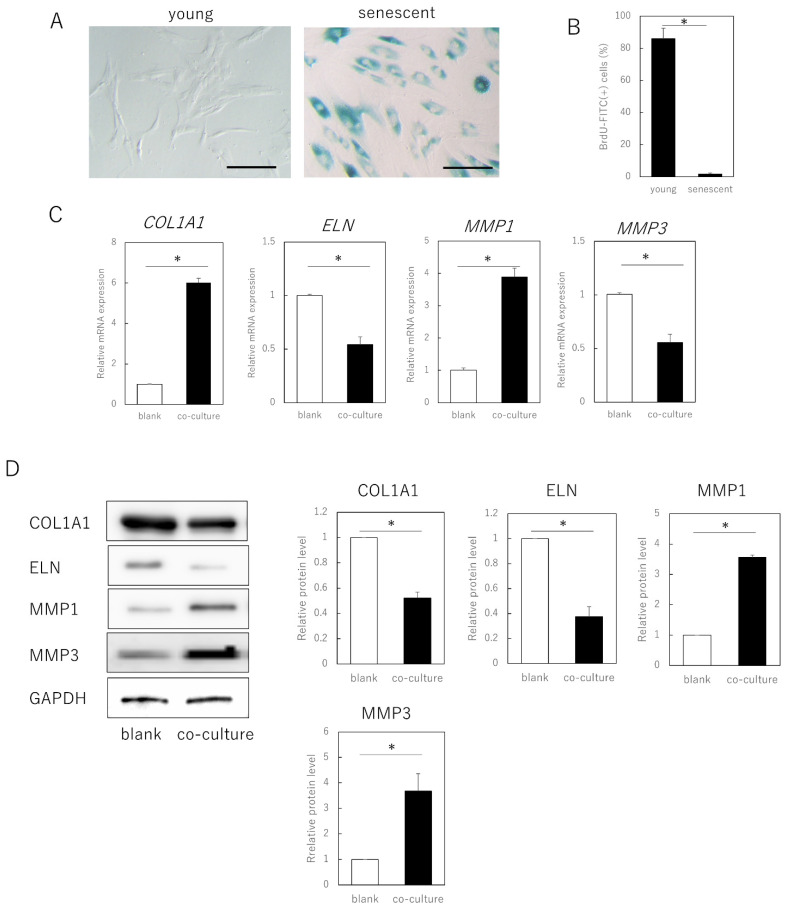
Expression of extracellular matrix-related genes in senescent dermal fibroblasts. (**A**) SA-β-gal staining of proliferating and senescent cells. Bar = 100 µm. (**B**) BrdU absorption in proliferating and senescent cells. (**C**) RT-qPCR analysis of extracellular matrix-related gene expression in young and senescent cells. *GAPDH* was used as a reference gene. (**D**) Western blot analysis of extracellular matrix-related protein expression in young and senescent cells. The expression levels of each protein were normalized to the expression level of GAPDH. * *p* < 0.05. All assays were conducted in experimental triplicate.

Senescent cells were obtained by long-term culture (>3 months, population doubling level [PDL] ≥ 50) of adult human skin fibroblasts and exhibited a typical senescent cell phenotypic features, including cytoplasmic expansion, SA-β-galactosidase expression, and decreased division (*p* = 0.00092) (Figure 1A,B). When these cells were co-cultured with young fibroblasts (PDL ≤ 30) in a semipermeable membrane separation system, gene expression of type I collagen α 1 chain (*COL1A1*, *p* = 0.00021) and elastin (*ELN*, *p* = 0.00034) in the young cells was significantly decreased, whereas that of matrix metalloproteinase (*MMP*)*1* (*p* = 0.00022) and *MMP3* (*p* = 0.000019) was significantly increased (Figure 1C). No significant changes occurred when young fibroblasts were co-cultured (data not shown).

Consistent with the gene expression results, at the protein level, young cells co-cultured with aged cells also showed decreased expression of COL1A1 (*p* = 0.00021) and ELN (*p* = 0.00033), and increased expression of MMP1 (*p* = 0.00017) and MMP3 (*p* = 0.0035). As the cells could not pass through the semipermeable membrane, aged fibroblasts negatively affected fibroblast matrix-related gene expression in young cells through secreted factors, rather than by direct cell contact.

### 3.2. EPDR1 Mediates the Negative Effects of Aging Skin Fibroblasts on Young Fibroblasts

**Figure 2:**
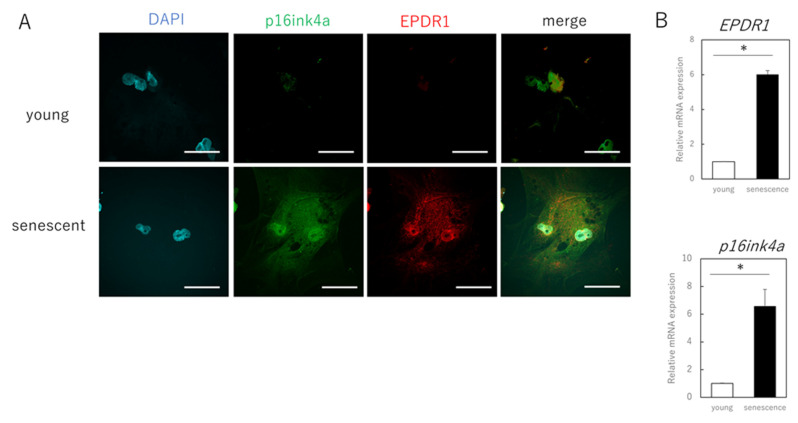

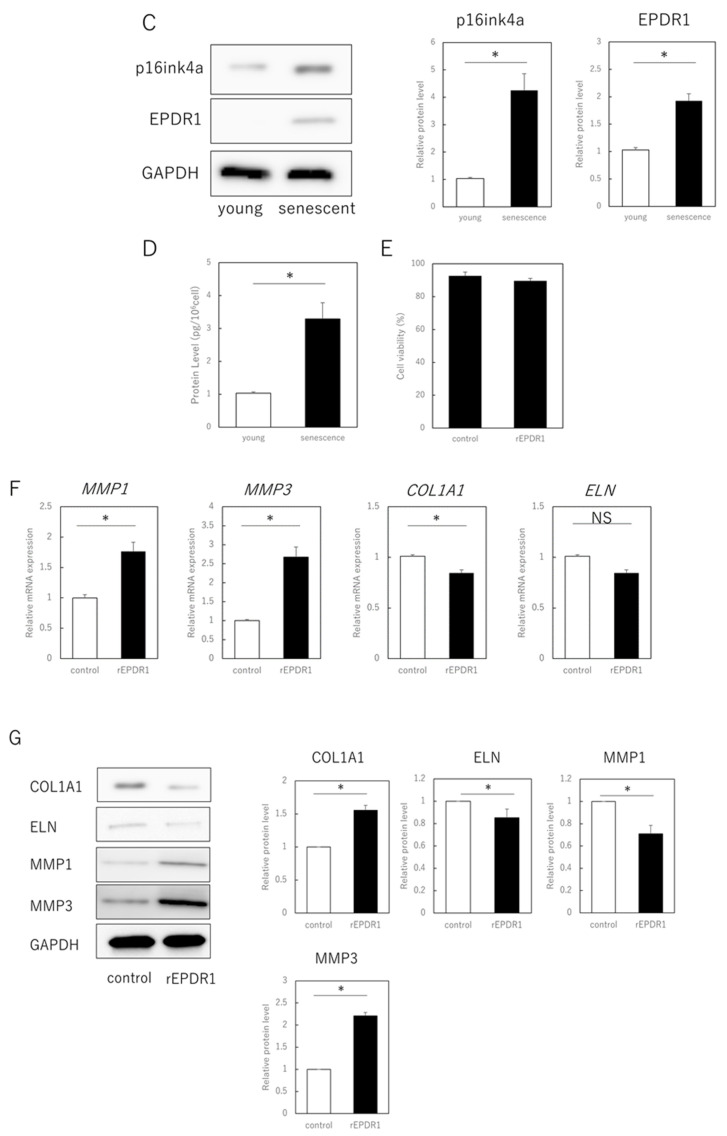

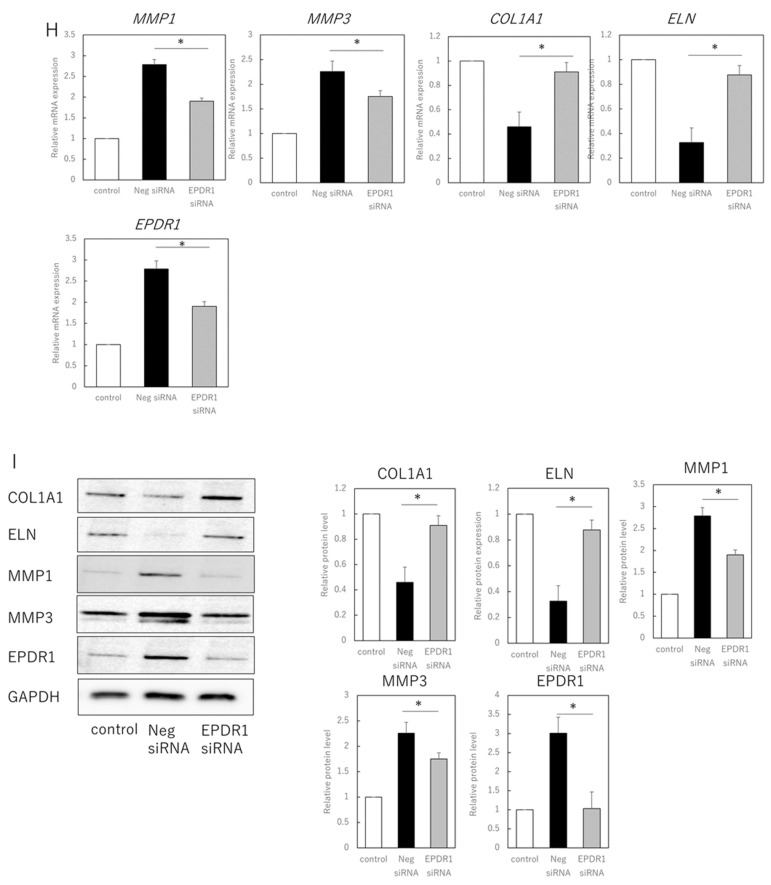
EPDR1 expression in aged dermal fibroblasts. (**A**) Immunostaining of p16ink4a and EPDR1 in young (proliferating) and senescent cells. Merge is a combined fluorescence image of DAPI, p16ink4a, and EPDR1. Bar = 20 µm. (**B**) RT-qPCR analysis of *p16ink4a* and *EPDR1* gene expression. *GAPDH* was used as a reference gene. (**C**) Western blot analysis of EPDR1 expression in young and senescent cells. GAPDH was used as a loading control. (**D**) ELISA of EPDR1 protein levels in culture medium of senescent cells. (**E**) Cell viability after recombinant EPDR1 treatment. (**F**) RT-qPCR analysis of changes in gene expression following the administration of recombinant EPDR1. *GAPDH* was used as a reference gene. (**G**) Western blot analysis of protein expression after recombinant EPDR1 treatment. The expression levels of each protein were normalized to the expression level of GAPDH. (**H**) RT-qPCR analysis of changes in gene expression after *EPDR1* knockdown. *GAPDH* was used as a reference gene. (**I**) Western blot analysis of protein expression after *EPDR1* knockdown. The expression levels of each protein were normalized to the expression level of GAPDH. * *p* < 0.05. All assays were conducted in experimental triplicate.

The next step was to identify the secreted factors from aging cells that affected extracellular matrix metabolism in young cells. We focused on EPDR1 and found that its gene and protein expression were increased in senescent cells (Figure 2A–C). ELISAs confirmed that the concentration of EPDR1 protein in the culture medium of aged fibroblasts was significantly increased (*p* = 0.0023) (Figure 2D). To examine the effect of EPDR1, human recombinant EPDR1 was added to young dermal fibroblast cultures. This did not affect cell viability (*p* = 0.68) (Figure 2E), but in accordance with the changes observed in the co-culture system of aged and young fibroblasts, *MMP1* (*p* = 0.00022) and *MMP3* (*p* = 0.000028), expression was increased in young fibroblasts treated with recombinant EPDR1, whereas *COL1A1* expression was decreased (*p* = 0.000031) (Figure 2F). *ELN* expression tended to be decreased, albeit not significantly (*p* = 0.12). However, at the protein level, there was an increase in MMP1 (*p* = 0.00022) and MMP3 (*p* = 0.000017), and a decrease in COL1A1 (*p* = 0.00038) and ELN (*p* = 0.0012) expression, all of which were significant (Figure 2G).

SiRNA-mediated knockdown of *EPDR1* in aged fibroblasts decreased *MMP1* (*p* = 0.00023) and *MMP3* (*p* = 0.000339) expression and increased *COL1A1* expression (*p* = 0.000018) (Figure 2H). This was confirmed using another EPDR1 siRNA with a different sequence. These results were also consistent at the protein level, with a decrease in MMP1 (*p* = 0.0013) and MMP3 (*p* = 0.00021), and an increase in COL1A1 (*p* = 0.0019) and ELN (*p* = 0.031) expression (Figure 2I).

### 3.3. EPDR1 Expression Is Increased in the Dermis of Aged Human Skin

**Figure 3:**
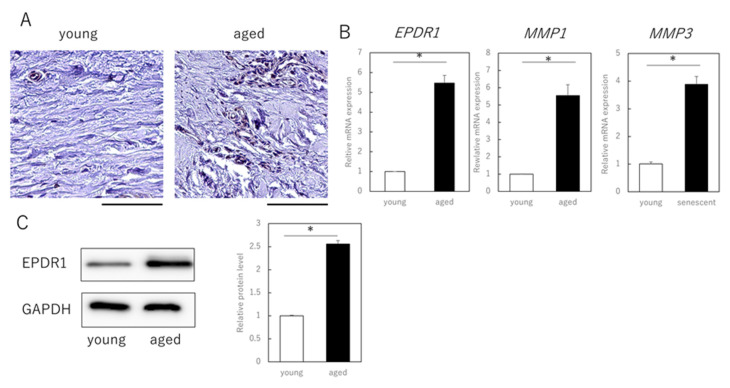
EPDR1 expression in aged human skin. (**A**) Immunostaining of EPDR1 in human skin dermis. (**B**) RT-qPCR analysis of gene expression using total RNA extracted from human skin dermis. *GAPDH* was used as a reference gene. (**C**) Western blot analysis using proteins extracted from human skin dermis. * *p* < 0.05. All assays were conducted in experimental triplicate.

Finally, we examined whether EPDR1 is actually expressed in dermal layers of aged (89-year-old) and young (3-year-old) individuals. Immunohistochemistry confirmed that EPDR1 was increased in aged dermis, compared to young dermis (Figure 3A). *EPDR1* expression was significantly higher in aged dermis than in young dermis (*p* = 0.00017), as was *MMP1* (*p* = 0.00033) and *MMP3* (*p* = 0.00011) expression (Figure 3B). Similar changes were observed at the protein level (*p* = 0.00017) (Figure 3C).

## 4. Discussion

To confirm the hypothesis that EPDR1, which is involved in cellular metabolic derangement and collagen deposition, functions as one of the secreted factors that cause cellular senescence, we observed co-cultures of senescent cells with young cells and investigated changes in the senescence phenotype caused by external intervention of EPDR1 expression.

Our results show that EPDR1 secreted by aging dermal fibroblasts negatively affects matrix-related gene expression in nearby young dermal fibroblasts. In addition, we found that EPDR1 increased matrix-related gene expression in both aged and young fibroblasts. The knockdown of *EPDR1* significantly blocked the increase in MMP-related gene expression. The in vitro findings, related to EPDR1, were also observed in human skin dermis samples. Overall, our findings indicate that EPDR1 plays at least a partial role in mediating age-dependent changes in dermal matrix status.

Elastin and type I collagen decrease in aging dermis because of decreased synthesis and increased degradation [27]. In this study, aging cells reduced *ELN* and *COL1A1* expression levels in surrounding fibroblasts. Exogenous EPDR1 supplementation decreased the expression of these genes, whereas siRNA-mediated knockdown increased their expression.

The *EPDR1* transcript is translated into proteins containing transmembrane domains and/or ependymin domains, and the association of ependymin with collagen demonstrates that these secreted proteins bind to collagen [28,29]. We propose the hypothesis that the EPDR1 protein, which contains transmembrane and ependymin domains, promotes collagen attachment by binding collagen via the ependymin domain, while remaining anchored to the cell via the transmembrane domain. This hypothesis is supported by previous findings that the knockdown of *EPDR1* in a Dupuytren’s contracture model, delayed and modestly attenuated contractility [30]. Furthermore, MMP1 a type I collagen-degrading enzyme, is induced and increased in fibroblasts of aging dermis [31]. Thus, aging cells may contribute to an age-dependent decrease in type I collagen by inducing MMPs via EPDR1 secretion, leading to deterioration of the dermal matrix condition. However, additional studies are needed to understand the biomolecular complexity of the EPDR1 protein in skin aging, as EPDR1 signaling itself is largely unexplored.

Targeting the SASP involving EPDR1 may be an important strategy for controlling the adverse effects of senescent cells. MMPs secreted by senescent cells may chronically impair skin conditions but contribute to tissue remodeling or hypertrophy during wound healing [32]. Thus, the SASP may have both positive and negative aspects; a better understanding of the SASP, and its effects on intracellular signaling in target cells, is required to support the development of specific drugs for aging-related diseases.

The limitation of this study was that the mechanism, by which manipulation of *EPDR1* expression affects the aging phenotype, was not determined. Future studies should examine the effects of EPDR1 in adults using a knockout mouse model. In addition, our results suggest that EPDR1 is effective in human skin fibroblasts and keratinocytes and may contribute to the development of skin rejuvenation therapies, however, no conclusions can be drawn regarding the aging of other cell types or fibroblasts of other tissues. If EPDR1 proves to be useful in fibroblasts of other tissues as well, it could contribute to the development of therapies to eliminate senescent cells in other organs. Finally, it will be necessary to test and assess EPDR1 expression in other models of senescence (e.g., oncogene-induced senescence [33] or D-galactose (D-Gal)-induced senescence [34]) and tumors when considering its application in systemic organs. Since human skin and rodents, including mice, are different in nature, the previously used human-chimeric mouse model should be employed to observe the phenotype of EPDR1 intervention on skin [35].

In conclusion, our results support that senescence-associated aged skin fibroblasts adversely affect surrounding fibroblasts via secreted factors. One such factor is EPDR1, which may represent a therapeutic target for the regulation of aging-related skin disorders.

## Data Availability

The data that support the findings of this study are available from the corresponding author, KT, upon reasonable request.
